# Proteins in Autophagic Machinery

**DOI:** 10.3390/cells10081987

**Published:** 2021-08-05

**Authors:** Ricardo Escalante

**Affiliations:** Instituto de Investigaciones Biomédicas Alberto Sols, CSIC/UAM, 28029 Madrid, Spain; r.escalante@csic.es or rescalante@iib.uam.es

Autophagy is a conserved self-degradation process that is activated under a wide variety of stresses and physiological conditions. The hallmark of autophagy is the formation of a double membrane vesicle, called the autophagosome, that envelops cellular material and delivers it to the lysosomes, where degradation takes place, and simple compounds are released into the cytoplasm for recycling or energy production. The Special Issue “Proteins in Autophagic Machinery”, published by *Cells*, includes three excellent reviews covering different aspects of the autophagy process, including autophagy in protists, the role of BECLIN1 protein, and the importance of selective autophagy in spermatogenesis and male fertility. These are clear examples of the conservation of the autophagic machinery during evolution, the complexity of its regulation, and the impact of autophagy on human health.

The degradation of cellular components by autophagy is an essential process for cell homeostasis and survival. Autophagosome biogenesis is regulated by the so-called autophagic machinery, a series of proteins (ATG proteins) organized in complexes that function in a hierarchical manner. The initiation step is regulated by the Atg1/ULK and PI3KC3 kinase complexes. The Atg1/UlK complex consists of Atg1, Atg13, Atg17, Atg29, and Atg31 in the yeast model *S. cerevisiae*, whereas in mammalian cells, it is composed of ULK1/2, ATG13, ATG101, and FIP200 [1]. The PI3KC3 complex is formed by Vps15, Vps34, Atg6, and Atg14 in *S. cerevisiae* and VPS15, VPS34, BECLIN1, NRFB2, and ATG14 in mammalian cells [1]. The generation of PtdIns3P by the class 3 lipid kinase VPS34 enables the recruitment of PROPPIN proteins (beta-propellers that bind polyphosphoinositides), which together with ATG2 (Atg2 in *S. cerevisiae* and ATG2A/ATG2B in mammals) are proposed to be responsible for the elongation of the autophagosome membrane originating from the fusion of ATG9-containing vesicles [2,3,4,5]. Lipids involved in membrane elongation are transported from subregions of the endoplasmic reticulum containing VMP1 and TMEM41B. These two proteins, as well as ATG9, have scramblase activity that is necessary to equilibrate the lipid bilayer of the ER and phagophore [5,6,7]. One critical feature of the autophagosome membrane is the covalent lipidation of the ATG8 protein that requires a conserved set of conjugation reactions performed by Atg3, Atg5, Atg7, Atg12, and Atg16 [8].

Autophagy is a highly conserved process in eukaryotes, including protists, a very large and phylogenetically diverse group of eukaryotic organisms with diverse lifestyles. Among them, parasitic protists are particularly interesting because of their relevance in pathology. They are responsible for human diseases such as leishmaniasis, trypanosomiasis, malaria, toxoplasmosis, etc. In their review, Sukamoto et al. analyze the repertoire of core autophagy proteins in parasitic protists and compare it with that of the opisthokonta group (fungi and Metazoa) [9]. The authors suggest that the autophagic machinery, or at least part of it, may have been present in the primitive common eukaryote ancestor known as LECA. One conclusion of this comparative study is that although proteins of the autophagic machinery have been partially conserved in protists, a clear reduction is observed in parasitic protists, which may be a consequence of specific adaptations to a parasitic life [9]. A clear example is the reduction observed in the parasitic amoeba *E. histolytica* compared to the non-pathogenic amoeba *D. discoideum* [10]. Interestingly, the least conserved proteins are those of the ATG1 complex. The authors also review the role of autophagy in different groups of protists. It is notable that, in some cases, certain autophagic proteins appear to play non-canonical roles in cell differentiation and organelle function. A notable example is the apicoplast, a specific plastid present in the apicomplexa group that requires the function of several ATG proteins for its biogenesis.

BECLIN1 is a complex protein involved in a wide variety of functions, including autophagy and other membrane trafficking processes. It is a conserved scaffolding subunit of the PI3KC3 complexes which, together with VPS34 and VPS15, forms the common core of the C1 and C2 complexes. While complex C1 contains ATG14 and is dedicated to autophagy, complex C2 contains UVRAG and regulates other membrane trafficking processes. Tran et al. comprehensively review the different aspects of this complex protein, including structural aspects, interactors, and function [11]. The authors discuss the interaction of BECLIN1 with BCL-2, as a remarkable example of crosstalk between autophagy and apoptosis. The authors also discuss phosphorylation and ubiquitination events in BECLIN1 that are linked to autophagy activation or inhibition, which add another layer of complexity in the regulation of autophagy.

Selective autophagy targets specific materials for degradation, such as damaged organelles, misfolded proteins, or pathogens. Recognition of different cargoes requires the function of multiple proteins, called autophagy receptors, that recognize cargoes and regulate the core autophagic machinery. The review by Lv et al. discusses the role of selective autophagy in reproduction, specifically in spermatogenesis and fertilization [12]. The authors highlight the role of mitophagy, lipophagy, and ER-phagy and the proteins involved in these forms of selective autophagy in the regulation of spermatogenesis. This process of cell differentiation requires the removal of part of the mitochondria by autophagy. Based on the published literature, Lv et al. propose that after fertilization, paternal mitochondria are ubiquitinated and targeted for selective degradation by autophagy, and thus, autophagy could be a therapeutic target to treat male infertility. The authors also discuss the role of lipophagy in providing energy for spermatogenesis.
